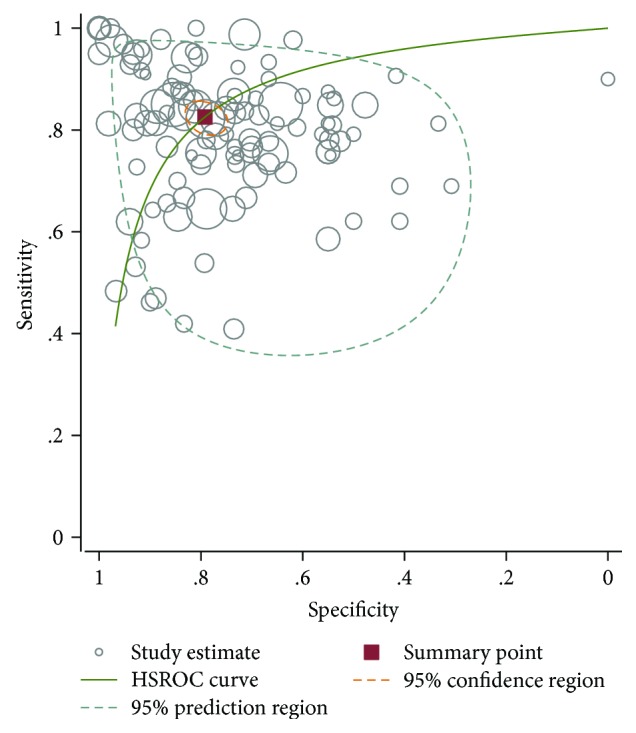# Response to: Comment on “Systematic Review and Meta-Analysis of Diagnostic Accuracy of miRNAs in Patients with Pancreatic Cancer”

**DOI:** 10.1155/2019/6287315

**Published:** 2019-02-05

**Authors:** Xiao Sun, Xiaobin Zhou, Haihua Liu

**Affiliations:** Department of Epidemiology and Health Statistics, College of Public Health, Qingdao University, Qingdao, Shandong 266021, China

Recently, we received the letter to the editor [1] in response to our recently published paper titled “Systematic Review and Meta-Analysis of Diagnostic Accuracy of miRNAs in Patients with Pancreatic Cancer [2].” We appreciate the interest and comments from Jayaraj et al. regarding our work. However, there are some issues in the comments that need to be addressed.

Regarding the differences between this study and previous publications [3–5], we wish to elaborate on the following: (1) 109 studies are included in this meta-analysis, which was more than the 9 studies in Wan et al. [3], the 52 studies in Ding et al. [4], and the 36 studies in Pei et al. [5]; (2) More subgroup analyses than in those articles [3–5] were performed, including race, source of control, miRNA profiling, and the combination of miR-21. In particular, subgroup analyses by the combination of miR-21 were not involved in the previous three articles. We found that miR-21s as biomarkers for the early diagnosis of PaC were more valuable than other miRNAs; (3) Our review conducted metaregression analyses to explore potential sources of heterogeneity and to confirm the results of subgroup analyses, but Wan et al. [3] and Pei et al. [5] did not. A large sample and more suitable statistical analysis method allow our study to provide more accurate and reliable information for future studies.

The diagnostic cut-off point plays an important role in disease diagnosis. There is not a singular diagnostic cut-off point for different miRNA profiling and source of miRNA. We originally prepared our subgroup analysis and sensitivity analysis based on different miRNA profiling and source of miRNA, but half of the included studies did not report the testing threshold. Therefore, such subgroup analysis and sensitivity analysis cannot be performed, which may have an impact on the results. It was recommended that diagnostic test articles should report thresholds to provide raw information for future meta-analysis.

The analysis of diagnostic threshold was performed in our meta-analysis. Spearman's correlation coefficient was 0.147 (*P* = 0.127), suggesting that there is no threshold effect. Then an I-square parameter was adopted to estimate the heterogeneity between the studies. However, we agree with Jayaraj et al.'s suggestion that a Tau-squared statistical parameter might be suitable for being the estimated variation of heterogeneity between the effects for test accuracy observed in different studies. For the meta-analysis of diagnostic test accuracy (DTA), the hierarchical summary receiver operating characteristic (HSROC) allows both the existence of threshold effects and the heterogeneity between studies. We subsequently conducted HSROC and found that the pooled SEN was 0.82 (95% CI, 0.79–0.85) and the pooled SPE was 0.79 (95% CI, 0.75–0.82) (Figure 1) and basically consistent with the results of the original study, which also shows that our results are reliable and credible.

The Cochrane Handbook for Systematic Reviews explicitly mentions not to use methods like the Begg or Egger tests for publication bias of DTA and argues that it is best to use the test proposed by Deeks [6]. Applying such tests for funnel plot asymmetry in systematic reviews of DTA may often cause publication bias being incorrect [7]. Deeks' tests should be preferred to assess publication bias in DTA meta-analyses [8].

## Figures and Tables

**Figure 1:**